# Clinical Outcome and Drug Expenses of Intravitreal Therapy for Diabetic Macular Edema: A Retrospective Study in Sardinia, Italy

**DOI:** 10.3390/jcm10225342

**Published:** 2021-11-16

**Authors:** Chiara Altana, Matthew Gavino Donadu, Stefano Dore, Giacomo Boscia, Gabriella Carmelita, Stefania Zanetti, Francesco Boscia, Antonio Pinna

**Affiliations:** 1 Hospital Pharmacy, Azienda Ospedaliero Universitaria di Sassari, 07100 Sassari, Italy; chiara.altana@aousassari.it (C.A.); gabriella.carmelita@aousassari.it (G.C.); 2 Department of Biomedical Sciences, University of Sassari, 07100 Sassari, Italy; zanettis@uniss.it; 3 Ophthalmology Unit, Azienda Ospedaliero Universitaria di Sassari, 07100 Sassari, Italy; stefanodore@hotmail.com (S.D.); apinna@uniss.it (A.P.); 4 Department of Medical, Surgical and Experimental Sciences, University of Sassari, 07100 Sassari, Italy; bosciagiacomo@gmail.com; 5 Section of Ophthalmology, Department of Basic Medical Science, Neuroscience and Sense Organs, University of Bari, 70124 Bari, Italy; francescoboscia@hotmail.com

**Keywords:** diabetic macular edema, intravitreal agents, best corrected visual acuity, central retinal thickness, adverse drug reactions, intravitreal drug expenses

## Abstract

Background: Diabetic macular edema (DME) is a leading cause of visual loss in working-age adults. The purpose of this retrospective study was to perform an epidemiological analysis on DME patients treated with intravitreal drugs in a tertiary hospital. The clinical outcome, adverse drug reactions (ADRs), and intravitreal drug expenses were assessed. Methods: All DME patients treated with Ranibizumab, Aflibercept, Dexamethasone implant, and Fluocinolone Acetonide implant at the Sassari University Hospital, Italy, between January 2017 and June 2020 were included. Central macular thickness (CMT) and best corrected visual acuity (BCVA) were measured. ADRs and drug expenses were analyzed. Results: Two-hundred thirty-one DME patients (mean age: 65 years) received intravitreal agents. Mean CMT and BCVA were 380 μm and 0.5 LogMAR at baseline, 298 μm and 0.44 logMAR after one year (*p* = 0.04), and 295 μm and 0.4 logMAR at the end of the follow-up period. A total of 1501 intravitreal injections were given; no major ADRs were reported. Treatment cost was €915,000 (€261,429/year). Twenty non-responders to Ranibizumab or Aflibercept were switched to a Dexamethasone implant. In these patients, mean CMT and BCVA were 468 µm and 0.5 LogMar at the time of switching and 362 µm and 0.3 LogMar at the end of the follow-up (*p* = 0.00014 and *p* = 0.08, respectively). Conclusion: Results confirm that Ranibizumab, Aflibercept, and Dexamethasone implant are effective and safe in DME treatment. A switch to Dexamethasone implant for patients receiving Aflibercept or Ranibizumab with minimal/no clinical benefit should be considered.

## 1. Introduction

Diabetic macular edema (DME) is one of the most severe complications of diabetic retinopathy and the main reason for legal blindness among working-age individuals in developed countries [1,2].

Population-based studies have reported DME prevalence rates of 4.2% to 7.9% in type 1 diabetic patients and 1.4% to 12.8% in type 2 diabetic patients [3]. In a Cochrane review of the DME prevalence evaluated using optical coherence tomography (OCT), the prevalence rates covered a wider range (19–65%) [4].

Worldwide, the prevalence rate for diabetic retinopathy has been estimated at 34.6% (93 million people) [5]. In the U.S., the prevalence rate for retinopathy for all diabetic patients aged ≥40 years has been reported to be 28.5% (4.2 million people) [5]. The prevalence of diabetic retinopathy increases with increasing duration of disease [6].

In the Wisconsin Epidemiologic Study of Diabetic Retinopathy, the four-year incidence of diabetic retinopathy was 59% when age at diagnosis was <30 years [7]. Conversely, when age at diagnosis was ≥30 years, the incidence rate was 47% in insulin users and 34% in nonusers of insulin [8].

In diabetic retinopathy, structural changes of the retinal vascular network can be observed, leading to accumulation of fluids in the macular region, disruption of the blood-retinal barrier, and expression of various inflammatory factors, including the vascular endothelial growth factor (VEGF), intercellular adhesion molecules (ICAM-1), monocyte chemoattractant protein (MCP-1), interleukine-6, and others [9,10]. Recently, experimental and clinical evidence have shown that in addition to microvascular changes and inflammation, retinal neurodegeneration may contribute to retinal damage in the early stages of diabetic retinopathy [11]. In the most advanced stages, a proliferative diabetic retinopathy can occur, which may result in a vitreous hemorrhage and/or tractional retinal detachment [12,13]. 

In patients with DME, anti-VEGF agents and corticosteroids are the gold standard of therapy. The purpose of this study was to carry out an epidemiological analysis on DME patients treated with intravitreal drugs (anti-VEGF agents and corticosteroid-based implants) between January 2017 and June 2020 at the Sassari University Hospital, Northwest Sardinia, Italy. Specifically, the clinical outcome, therapy adherence, and drug expenses were assessed.

## 2. Materials and Methods

### 2.1. Participants

All 231 DME patients (139 men, 92 women; mean age: 65 years) treated with intravitreal drugs at the Ophthalmology Unit—Azienda Ospedaliero-Universitaria, Sassari, Italy, between January 2017 and June 2020 were included in this retrospective study.

Our unit has a catchment population of approximately 335,000 living in an area of 4300 square kilometers (Sassari province).

Ethical approval was waived by the local Ethics Committee of the Azienda Ospedaliero-Universitaria di Sassari in view of the retrospective nature of the survey, which was conducted in full accord with the tenets of the Declaration of Helsinki. Each participant received detailed information and provided informed consent.

Affected eyes received a loading dose of three consecutive monthly intravitreal injections of Ranumizumab (0.3 mg) or five consecutive monthly injections of Aflibercept (2.0 mg), followed by a treat-and-extend regimen. This regimen incorporates elements of both monthly and as-needed (PRN) treatment regimens. As with a monthly regimen, the ophthalmologist administers anti-VEGF intravitreal injections at each follow-up examination, but instead of a fixed 4-week follow-up interval, the length of the interval varies according to disease activity.

In patients presenting a serous detachment of neuroepithelium and/or a poor response in terms of improvement of best corrected visual acuity (BCVA) and central macular thickness (CMT) three months after the loading phase with Ranimizumab or Aflibercept, intravitreal therapy was switched to a Dexamethasone implant (700 µg), administered twice yearly, or to a Fluocinolone Acetonide implant (190 µg).

### 2.2. Data Analysis

Data regarding treatments were extracted from the Eye Clinic records, web-based monitoring records by the Italian Medicines Agency (Agenzia Italiana del Farmaco-AIFA), and data flows included in the New Health Information System (Nuovo Sistema Informativo Sanitario—NSIS).

Data about the treatment period (months) and regimen were extracted from AIFA web-based monitoring records. In addition, the analysis also yielded information regarding the number and types of therapy switches during the period under analysis. Furthermore, the study verified adherence of treatments to therapy protocols, assessing the number of injections performed.

Evaluation of treatment efficacy was based on the measurement of CMT expressed in μm and BCVA expressed in logMAR. CMT data were obtained by using Topcon OCT 2000 (Japan). The analysis compared clinical parameters at baseline, after the first year, and at the end of the follow-up period. Evaluation of data on efficacy was carried out only for patients who received at least one year of therapy. 

Data on safety were evaluated as the number and severity of suspected adverse reactions to drugs (Adverse Drug Reactions, ADRs) observed during analysis of clinical documents.

Costs of intravitreal drugs under analysis were extracted by the IT System of the Sardinian Region adopted by all healthcare facilities in Sardinia.

For drugs not monitored by AIFA (corticosteroids), the epidemiological analysis was conducted on data obtained from the patients’ clinical records.

### 2.3. Statistical Analysis 

Qualitative data are shown as number and percentages. The analyzed data showed a normal distribution (Kolmogorov–Smirnov test); hence, the Student’s *t*-test for continuous variables was used. A *p*-value < 0.05 was considered statistically significant. The analysis was carried out by using IBM SPSS Statistics 24 for Windows.

## 3. Results

The number of patients with bilateral DME was 78 (34%) out of 231. The total number of DME eyes treated with intravitreal drugs was 309. Each eye received on average 4.5 intravitreal injections per year. Overall, the total number of intravitreal injections administered for DME treatment during the period under analysis was 1501, with an average of 375 injections per year. The most used agent was Ranibizumab (46%), followed by Aflibercept (34%) and Dexamethasone implant (20%), while Fluocinolone Acetonide intravitreal implants accounted for less than 1% of administrations (Table 1). Dexamethasone and Fluocinolone Acetonide implants were never used as first line therapy.

Adherence to therapy was evaluated as number of interruptions before the end of the loading phase (three doses with 4-week intervals for Ranibizumab and five doses with 4-week intervals for Aflibercept). The rate of interruptions was 12%, with 10% represented by Ranibizumab. During the analyzed period, a total of 34 therapy switches occurred. Ranibizumab or Aflibercept was switched to a Dexamethasone implant in 20 (59%) cases, whereas a switch to a Fluocinolone Acetonide implant was performed in two (6%).

**Figure 1:**
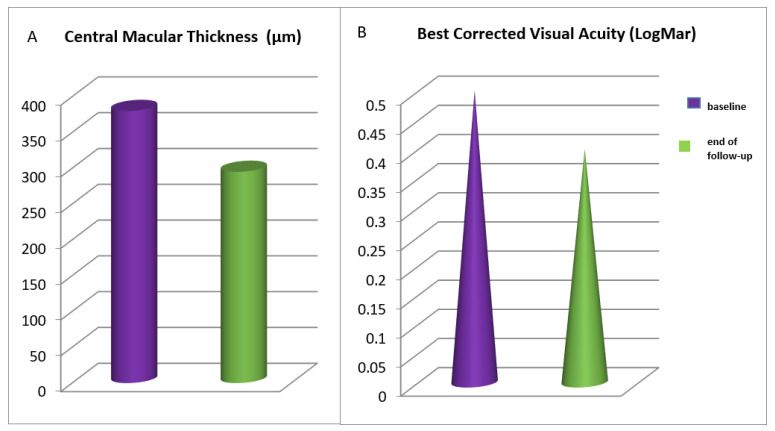
Central macular thickness (**A**) and best corrected visual acuity (**B**) values at baseline and at the end of the follow-up for all patients receiving intravitreal treatment for diabetic macular edema.

The mean CMT at baseline was 380 μm. After the first year of therapy, the mean CMT was 298 μm, with an 82 μm reduction (*p* = 0.04). At the end of the follow-up period, the mean CMT remained substantially unchanged (295 μm). The mean BCVA was 0.5 LogMAR at baseline, 0.44 logMAR after one year of treatment (*p* = 0.04), and 0.4 logMAR at the end of treatment. CMT and BCVA values for all patients are summarized in Figure 1.

**Figure 2:**
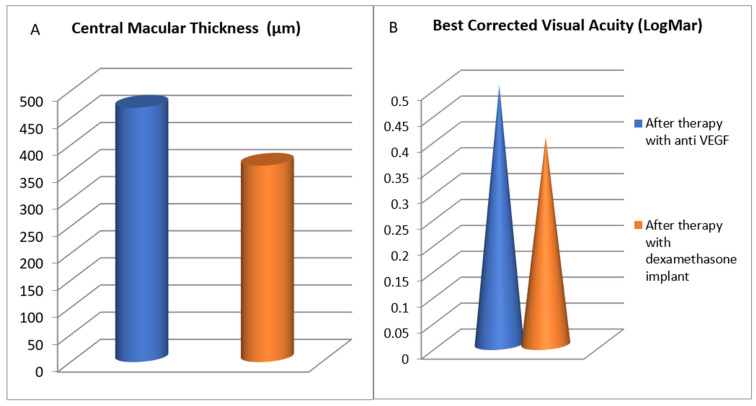
Central macular thickness (**A**) and best corrected visual acuity (**B**) values at the time of switching to a Dexamethasone implant and at the end of the follow-up.

In the 20 patients switched to a Dexamethasone implant, the mean CMT was 468 µm at the time of switching and 362 µm at the end of the follow-up, a statistically significant difference (*p* = 0.00014). The mean BCVA was 0.5 LogMar at the time of switching and 0.3 LogMar at the end of the follow-up, again a statistically significant difference (*p* = 0.08). CMT and BCVA values before and after the switch to Dexamethasone are shown in Figure 2.

Considering all types of retinal diseases for which intravitreal drugs are used (i.e., wet age-related macular degeneration, retinal vein occlusion, myopic choroidal neovascularization, etc.), the total treatment cost during the analyzed period amounted to €5,780,000 (Table 2), with an average cost of €1,651,429 per year. As far as DME is concerned, the global cost of intravitreal treatment was €915,000, with an average cost of €261,429 per year, accounting for approximately 16% of total expenses. It is important to emphasize that the total expense per year is subject to high variability, due to differences in drug consumption and selling price, with the latter decreasing over the years. Ranibizumab was the drug that mostly affected the expense, as it was the most prescribed intravitreal agent (46%), followed by Aflibercept (34%).

Our study showed that the analyzed treatments were generally well tolerated. Indeed, during the period taken into consideration, no major ADRs (endophthalmitis, occlusive vasculitis, etc.) were reported.

## 4. Discussion

DME represents a social burden due to the reduction of vision and lower quality of life of patients affected [1,2]. Our retrospective survey confirms the clinical efficacy of Ranibizumab, Aflibercept, and Dexamethasone implant in the treatment of DME [5,14,15], with significant BCVA improvement and CRT reduction, especially after the first year of treatment. In terms of safety, the drugs analyzed showed a good risk-benefits outcome, with no systemic ADRs. Occasionally, local reactions were reported, mostly related to the injection procedure and not connected to pharmacologic properties of the intravitreal agents. It is important to emphasize that no thromboembolic events were observed during the analyzed period. On the other hand, data related to adherence to therapy showed that as much as 12% of the patients did not complete the loading phase, with a negative impact on the outcome. The greatest contribution to this value was given by Ranibizumab, the most used anti-VEGF agent in this investigation. Unfortunately, from the available data, it was not possible to ascertain the reason of treatment interruption in most cases. 

In our study, 20 patients receiving intravitreal Ranimizumab or Aflibercept with disappointing results were switched to a Dexamethasone implant. After switching, there was a significant improvement in the mean BCVA (from 0.5 to 0.3 LogMar) and CMT (from 468 to 362 µm).

In patients presenting a serous detachment of neuroepithelium and poor response in terms of BCVA and CMT after the loading phase with Ranimizumab or Aflibercept, intravitreal injection of a Dexamethasone implant should be considered. This can be administered as an adjunctive treatment, without suspending the anti-VEGF agents, or as a switch treatment. 

Overall, expenses for DME treatment accounted for only 16% of the global cost of all intravitreal injections for any cause. This can at least in part be explained by the fact that about one-third of diabetic patients are not aware of having the disease; therefore, diabetic retinopathy and DME are commonly underdiagnosed [5]. Cost analysis did not show a linear trend over time, presenting a peak in 2018. In 2020, the expenses were considerably lower, as eye examinations and intravitreal injections fell due to the hospital emergency created by the first COVID-19 wave; furthermore, there was a reduction in the selling price of all the intravitreal agents used. 

Our study has important limitations, including its retrospective nature and the use of relatively crude estimates of hospital expenses. However, it provides some new data in an underinvestigated topic, such as the drug expenses of intravitreal treatment for DME.

## 5. Conclusions

In conclusion, our results confirm that Ranibizumab, Aflibercept, and Dexamethasone implant are effective and safe in the treatment of DME. A therapy switch to Dexamethasone implant for patients receiving Aflibercept or Ranibizumab with minimal/no clinical benefit is recommended in an attempt to improve vision, reduce costs, and reduce the burden of injections of clinics and hospitals, especially in a pandemic era [16].

## Figures and Tables

**Table 1:** Intravitreal agents used in the treatment of diabetic macular edema (DME).

Drug	Average Number (%) of Intravitreal Injections per Year
Ranibizumab	172 (45.85%)
Aflibercept	127 (34%)
Dexamethasone implant	76 (20%)
Fluocinolone Acetonide implant	2 (0.15%)
Bevacizumab	0%

**Table 2:** Annual number and cost of intravitreal (IV) injections in the period January 2017–June 2020.

Year	All IV Injections (n.)	Global IV Cost (€)	IV Injections for DME	IV Cost for DME * (€)
2017	2416	1,180,000.00€	358	175,000.00€
2018	2720	1,750,000.00€	388	250,000.00€
2019	2642	1,600,000.00€	643	390,000.00€
2020 ^†^	1395	1,250,000.00€	112	100,000.00€
TOTAL	9173	5,780,000.00€	1501	915,000.00€

* DME = Diabetic Macular Edema; ^†^ January–June.

## Data Availability

Data will be available upon request.
